# Borrelia Ecology and Evolution: Ticks and Hosts and the Environment

**DOI:** 10.3390/microorganisms10081513

**Published:** 2022-07-26

**Authors:** Gabriele Margos, Anna Jonsson Henningsson, Mateusz Markowicz, Volker Fingerle

**Affiliations:** 1 National Reference Center for Borrelia, Bavarian Health and Food Safety Authority, Veterinärstr, 2, 85764 Oberschleissheim, Germany; volker.fingerle@lgl.bayern.de; 2 Department of Biomedical and Clinical Sciences, Division of Inflammation and Infection, Linköping University, 581 83 Linköping, Sweden; anna.jonsson.henningsson@rjl.se; 3 Department of Clinical Microbiology in Jönköping, Linköping University, Region Jönköping County, 581 83 Linköping, Sweden; 4 Department of Clinical Microbiology in Linköping, Linköping University, 581 83 Linköping, Sweden; 5 AGES—Austrian Agency for Health and Food Safety, 1090 Vienna, Austria; mateusz.markowicz@meduniwien.ac.at

The genus *Borrelia* encompasses bacterial pathogens that can cause Lyme borreliosis (LB) and relapsing fever (RF). Additionally, apart from known pathogenic species, in recent years a great deal of diversity has been recognized to exist within the genus *Borrelia* [1]. The bacteria have an almost global distribution—*Borrelia burgdorferi* sensu lato occurs mainly in the temperate zones of Northern Hemisphere, whilst the majority of RF spirochetes occurs in subtropical and tropical regions, except for *B. miyamotoi*, an RF species that occurs in the temperate zones [2,3,4,5,6]. As a parasitic pathogen, Borrelia’s ecology and evolution is intimately intertwined with vector and host ecology [7,8,9]. These in turn are vulnerable to alterations in their natural environment introduced through human-induced environmental change that may lead to changes in (micro)habitat conditions. Some of these changes are likely to be positive, improving survival conditions for vector and reservoir host, but others may be negative, depleting tick and/or reservoir host populations. These impacts on tick or host abundance, *Borrelia* infection prevalence in ticks and *Borrelia* population alterations may result in stable or changing risks for human to acquire the bacteria and to develop LB or RF [7,10,11,12,13,14,15]. As the human population is growing and space requirements for humans and their habitations are increasing, contact between humans, ticks and borreliae will also increase [15]. There are still many open questions that need to be addressed surrounding this complex system. How do we obtain fast and accurate information on *Borrelia* prevalence in nature in an ever-changing world? What is the impact of environmental changes on the pathogenicity of *Borrelia*? How do interactions with *Borrelia* impact hosts and vectors? Can we improve clinical diagnosis of Lyme borreliosis?

In this Special Issue, we have brought together reports from different lines of research on *Borrelia,* especially its ecology, evolution and interaction with the environment. Included papers report on *Borrelia* species, *Borrelia* populations, tick–*Borrelia* interactions and epidemiological questions, as well as LB diagnostics. The contribution by Norte et al. reports on the population structure of *B. lusitaniae* and compares it with the distribution of the vector species *Ixodes ricinus* and *Ixodes inopinatus* [16]. The authors suggest that populations of *B. lusitaniae* are shaped—at least partially—by the distribution of vector species, although molecular identification of the two tick species was a problematic issue. Weck et al. [17] describe a novel *Borrelia* species detected in Brazil that belongs to the *B. burgdorferi* sensu lato complex and appears to be associated with cricetid rodents as (reservoir) hosts. The species, termed “*Candidatus* Borrelia paulista” Rp42, was identified by multilocus sequence typing of eight chromosomal housekeeping genes. Three papers investigate the presence/prevalence of *Borrelia* and other tick-borne pathogens in wild or domesticated animals and the impact of environmental changes: Wijnveld et al. [18] used dogs as sentinels to explore the presence of tick-borne pathogens, including *Borrelia*, in Austria, whilst Qiu et al. [19] explored the prevalence of *B. theileri* in wild and domestic animals in the Kafue ecosystem in Zambia. The latter study provides the first evidence of *B. theileri* in African wildlife and cattle in Zambia. Boyer et al. [20] investigated the impact of anthropogenic environmental changes on the presence of tick-borne pathogens in France. Another three papers concern *Borrelia*–tick interactions. Schwan et al. [21] present convincing data on transovarial transmission of *B. hermsii* by its vector *Ornithodorus hermsii*, an interaction between bacterium and vector that was previously thought to be the exception from the rule (see [22]). Springer et al. [23] investigate “*Borrelia* infections in aging ticks”, exploring the relationship of the bacteria in field-collected *I. ricinus* nymphs. The interesting results of early transcriptional changes in the midgut of *Ornithodoros* following infection with *B. duttonii* are presented by Schäfer et al. [24]. Numerous putative and uncharacterized sequences were found, demonstrating the complexity of the tick response. Hoffmann et al. [25] report on the complex shifts of microbiota in ticks infected with *B. burgdorferi*. Significant read abundance differences were noticed for *Pseudomonas* and *Wolbachia* in ticks infected with human pathogenic *B. burgdorferi*. The presence of *Borrelia* was more important than location of tick collection for shifts in microbiota. The authors suggest that data on microbiota may help improving diagnostics and therapeutic interventions.

Regarding Lyme borreliosis in human hosts, three interesting papers are presented: one presented by Raffetin and co-authors [26] describes “Perceptions, representations and experiences of patients presenting non-specific symptoms”; the other two [27,28] describe studies using multidisciplinary methods for diagnosis of suspected Lyme borreliosis. Raffetin et al. investigated 569 patients with follow-up at three and 12 months. A subset of these patients presented with confirmed Lyme borreliosis. The authors conclude that a multidisciplinary approach may improve accuracy in diagnosis. The second paper evaluated differential diagnosis and clinical outcome of antibiotic treatment in patients with possible or suspected Lyme neuroborreliosis. Patients presenting with possible neuroborreliosis responded better to therapy than patients presenting with suspected neuroborreliosis. The latter group had diverse clinical manifestations and comorbidities that complicated differential diagnosis. The study emphasizes the importance of CSF analysis in Lyme neuroborreliosis.

There is currently much effort being expended in recording the frequency of occurrence of ticks and whether they are infected with borreliae, as this directly affects the risk to humans of becoming infected. We believe the message of the papers in this collection is that in order to understand the significance of such data for both the dynamics of *Borrelia* in both vectors and reservoir hosts and of LB and RF in humans we need to know much more about the environmental context in which infections occur.
